# Spectrum (characteristics) of patients with chronic kidney disease (CKD) with increasing age in a major metropolitan renal service

**DOI:** 10.1186/s12882-017-0781-5

**Published:** 2017-12-28

**Authors:** Usman Mahmood, Helen G. Healy, Adrian Kark, Anne Cameron, Zaimin Wang, Rajitha Abeysekera, Wendy E. Hoy

**Affiliations:** 1 Kidney Health Services, Metro North Hospital and Health Service, Royal Brisbane & Women’s Hospital, Brisbane, QLD Australia; 2 NHMRC CKD.CRE & CKD.QLD, Brisbane, QLD Australia; 3 Centre for Chronic Disease, University of Queensland, Brisbane, Australia; 4 Toowoomba Hospital, Toowoomba, QLD Australia

**Keywords:** Heterogeneity, Chronic kidney disease, Age distribution, Clinical characteristics, Outcomes, Renal replacement therapy, Death, Progression, Hospitalization, CKD.QLD registry

## Abstract

**Background:**

Aim of our study is to describe, in people with CKD, the demographic and clinical characteristics and outcomes with increasing age. The prevalence of CKD in Western populations, where longevity is the norm, is about 10–15%, but how age influence different characteristics of patients with CKD is largely not known.

**Methods:**

One thousand two hundred sixty-five patients enrolled in the CKD.QLD registry at the Royal Brisbane and Women’s Hospital were grouped according to age at consent i.e. <35, 35–44, 45–54, 55–64, 65–74, 75–84, 85+ years age groups, and were followed till start of renal replacement therapy (RRT), death, discharge or the censor date of September 2015.

**Results:**

Age ranged from 17.6 to 98.5 years with medians of 70.1 and 69.9 years for males and females respectively: 7% were <35 years of age, with the majority (63%) >65 years old. The leading renal diagnoses changed from genetic real disease (GRD) and glomerulonephritis (GN) in the younger patients to renovascular disease (RVD) and hypertension (HTN) in older patients. With increasing age, there were often multiple renal disease diagnoses, more advanced stages of CKD, greater number of comorbidities, more frequent and more costly hospitalizations, and higher death rates. The rates of initiation of renal replacement therapy (RRT) rose from 4.5 per 100 person years in those age < 35 years to a maximum of 5.5 per 100 person years in 45–54 years age group and were lowest, at 0.5 per 100 person years in those >85 years. Mortality rates increased by age group from 1.3 to 17.0 per 100 person years in 35–44 year and 85+ year age groups respectively. Rates of hospitalization, length of stay and cost progressively increased from the youngest to eldest groups. Patients with diabetic nephropathy had highest incidence rate of RRT and death. The proportion of patients who lost more than 5mls/min/1.73m^2^ of eGFR during at least 12 months follow up increased from 13.3% in the youngest age group to 29.2% in the eldest.

**Conclusion:**

This is the first comprehensive view, with no exclusions, of CKD patients seen in a public renal specialty referral practice, in Australia. The age distribution of patients encompasses the whole of adult life, with a broader range and higher median value than patients receiving RRT. Health status ranged from a single system (renal) disease in young adults through, with advancing age, renal impairment as a component of, or accompanying multisystem diseases, to demands and complexities of support of frail or elderly people approaching end of life. This great spectrum demands a broad understanding and capacity of renal health care providers, and dictates a need for a wider scope of health services provision incorporating multiple models of care.

## Background

Chronic Kidney Disease (CKD) is a significant public health problem worldwide because of the poor outcomes experienced by patients and high associated costs [1–3]. It is the most common chronic disease in Australia with approximately 10% of adults (1.7 million) affected according to estimates by the Australian Institute of Health and Welfare [4] and a prevalence of 14% as per the AusDiab study [5]. Control of CKD is confounded by its heterogeneity; of patient demographics, etiology of disease, and number and type of complications and co-morbidities. Patients in whom CKD is not controlled experience excess morbidity and mortality, especially cardiovascular mortality [6–9]. Furthermore the cost of managing these poor health outcomes is high with renal replacement therapy (RRT) for end stage kidney disease (ESKD) alone costing a huge amount of money to health systems [2]. All of the opportunity of controlling CKD, once present, lies with slowing disease progression, preventing complications, especially cardiovascular complications, and preventing ESKD. Early detection, better understanding of disease mechanisms and personalized treatment of the CKD complex are all needed. However, there are paucity of data of the interactions of demographics, etiologies, complications and co-morbidities, including their impact on the postulated pathway of a self-perpetuating vicious cycle of fibrosis independent of the initial renal injury [10].

Furthermore, the weight of different components of the complex causal interactions is changing. Elderly people are the fastest growing population group [11] and elderly patients are characterized by different causes of kidney disease and higher co-morbidities than younger age groups [12]. Therapies based on evidence generated in younger age groups may therefore not be applicable to those of an older demographic. Furthermore personalized treatment of CKD in the elderly must be cognizant of likely interacting and coexisting health needs in a cohort who have accumulated deficits in multiple organs requiring further customizing of their bundle of care. Modification of drug selection and dosing is one example [13].

We aim to describe patient, kidney and co-morbid characteristics across age groups in a large and current population of the Chronic Kidney Disease in Queensland (CKD.QLD) cohort. CKD.QLD [14]. CKD.QLD, established in late 2009, is a statewide multidisciplinary research and practice program, conducted through a collaboration of academics and specialists in kidney care in the public health sector in Queensland. CKD.QLD encompasses a CKD registry involving all consenting patients referred to public renal practices (prior to renal replacement therapy - RRT) in Queensland, which is the third most populous Australian state/territory. CKD.QLD has identified some important differences between CKD.QLD registry patients (a pre-dialysis population) and those patients who have survived a renal death and are on RRT in Australia. Notably, the CKD.QLD cohort included older people with no gender difference, unlike the male-dominant RRT population, and has a higher percentage of patients with renal vascular disease and a lower percentage with diabetic nephropathy [15].

## Methods

This is a prospective observational cohort study of 1265 participants who were enrolled, with informed consent, in the CKD.QLD registry [15] at the Kidney Health Service, Royal Brisbane and Women’s Hospital (Metro North Hospital and Health Service), Queensland Health. Their characteristics were documented at baseline; they were followed longitudinally and, for the purpose of this description of outcomes, censored at commencement of renal replacement therapy (RRT), death, discharge or September 2015.

“Chronic Kidney Disease in Queensland (CKD.QLD) Registry Study” has Royal Brisbane and Women’s Hospital Human Research Ethics Committee approval - HREC Reference Number (MNHHS): HREC/15/QRBW/294 as of June 2015 (Previous HREC Reference Number (OHMR): HREC/10/QHC/41 from Nov 2010 to June 2015 University of Queensland Medical Research Ethics Number: 2011000029).

### Participant population

The study population is a group of 1265 patients managed by a single service provider, the Kidney Health Service (KHS), Metro North Hospital and Health Service (MNHHS), a specialty service in the publicly funded health system. The first participant gave informed consent to join the CKD.QLD cohort in May 2011. Participants were progressively enrolled at clinical consultations at different clinic locations, all under the umbrella of KHS, MNHHS. Patients were grouped according to age at consent into <35, 35–44, 45–54, 55–64, 65–74, 75–84 and 85+ years.

### Inclusion and exclusion criteria

All adult patients referred to the MNHHS KHS, not on RRT, and able to give informed consent, were offered enrolment in the CKD.QLD Registry. Patients were excluded if pediatric, already on RRT, or unable to give informed consent. Rates of patients declining Registry participation were less than 1%.

### Data collection and patient characteristics

Data were extracted from the clinical records of participants, both electronic and hardcopy, at the time point of consent and throughout the study period. Outcomes were sourced from a combination of these records and organisational datasets, including hospital records for each admission and ANZDATA (http://www.anzdata.org.au) for a comparative population of RRT patients. Data extractions included 1) Demographics – age, gender, weight, height and derived body mass index (BMI) 2) cause/s of chronic kidney disease (clinical diagnosis) as determined by the treating Nephrologist; these causes were grouped into seven categories in both genders i.e. glomerulonephritis (GN), genetic renal disease (GRD), loss of single kidney, diabetic nephropathy (DN), renovascular disease including hypertension, and categories of other and uncertain. We also recorded the number of renal diagnoses in all patients, both males and females. Some participants were accorded more than one cause for CKD by their treating nephrologist. 3) Stage of CKD (1, 2, 3A, 3B, 4 and 5) at the time of consent to the registry 4) The presence of specific clinically diagnosed co-morbidities were recorded: these included coronary artery disease, cerebrovascular disease, hypertension, peripheral vascular disease, diabetes, gout, obstructive sleep apnoea, chronic lung disease and cognitive impairment/dementia 5) Outcomes measured included death, start of RRT (cause specific), hospitalisation including length of stay, causes of specific hospitalisation, and changes in eGFR over the follow-up period.

Stage of CKD was calculated by using CKD-EPI formula for estimated glomerular filtration rate (eGFR) [16]. The outcome of death was recorded as the raw number of patients who died and the incidence of death per 100 person years of follow up was calculated. The outcome of initiation of RRT was recorded as the raw number of patients who started RRT, the incidence of RRT per 100 person years and the primary renal diagnosis in that patient. Rates of hospitalisation per 100 person years and the top 10 causes of hospitalisation were recorded by using diagnosis related group (DRGs) code set. Changes in eGFR were transformed to rate of change of eGFR over 12 months and categorised as progressive if declining by >5 ml/min/1.73m^2^, improved if there was gain of >5 ml/min/1.73m^2^ and stable if there was less than +/− 5 ml/min/1.73m^2^ of eGFR change over at least 12 months follow up.

## Results

### Age, gender, ethnicity and body mass index (BMI)

Age of patients in our cohort ranged from a minimum of 17.7 years to a maximum of 98.5 years. Majority of patients i.e. 63% (798/1265) in our study were over 65 years of age. Gender distribution was similar, 651 males and 614 females. Out of 1265 participants in our study, 804 patients had available data on ethnicity. Vast majority of study population (791/804) was Caucasian (98.3%). 13/804 (1.6%) patients were of aboriginal/indigenous descent. Table 1 demonstrates age groups, number of patients in each age group, mean ages, gender and BMI.

**Table 1:** Age, gender distribution and median (IQR) BMI stratified for age groups

Age group (years)	Number (n)	Females (n)	Males (n)	Mean age (years)	Standard deviation (SD)	Median (IQR) BMI (kg/m^2^)
<35	89	53	36	27.4	2.3	27.7 (23.4–32.6)
35–44	76	38	38	40.7	2.8	29.2 (25.5–35.6)
45–54	122	56	66	50.5	2.8	29.0 (25.5–35.0)
55–64	180	82	98	60.5	2.8	31.6 (27.3–38.1)
65–74	359	163	196	70.2	2.8	31.2 (27.1–36.0)
75–84	341	172	169	80.0	2.8	28.1 (24.8–31.9)
85+	98	50	48	87.8	2.4	26.0 (23.4–29.0)
Total	1265	614	651	66.2	16.5	29.2 (25.3–34.5)

Height ranged from 130.5 cm to 223.6 cm and weight ranged from 32.2 kg to 200.9 kg. Median BMI was either in ‘overweight’ or ‘obese’ category in all age groups (Table 1) but tended to be lowest in the most elderly age group (85+ years). This is consistent with findings from Kramer’s “BMI in an incident ESRD population” study [17].

### Etiology/primary renal diagnoses and number of renal disease diagnoses

**Fig. 1:**
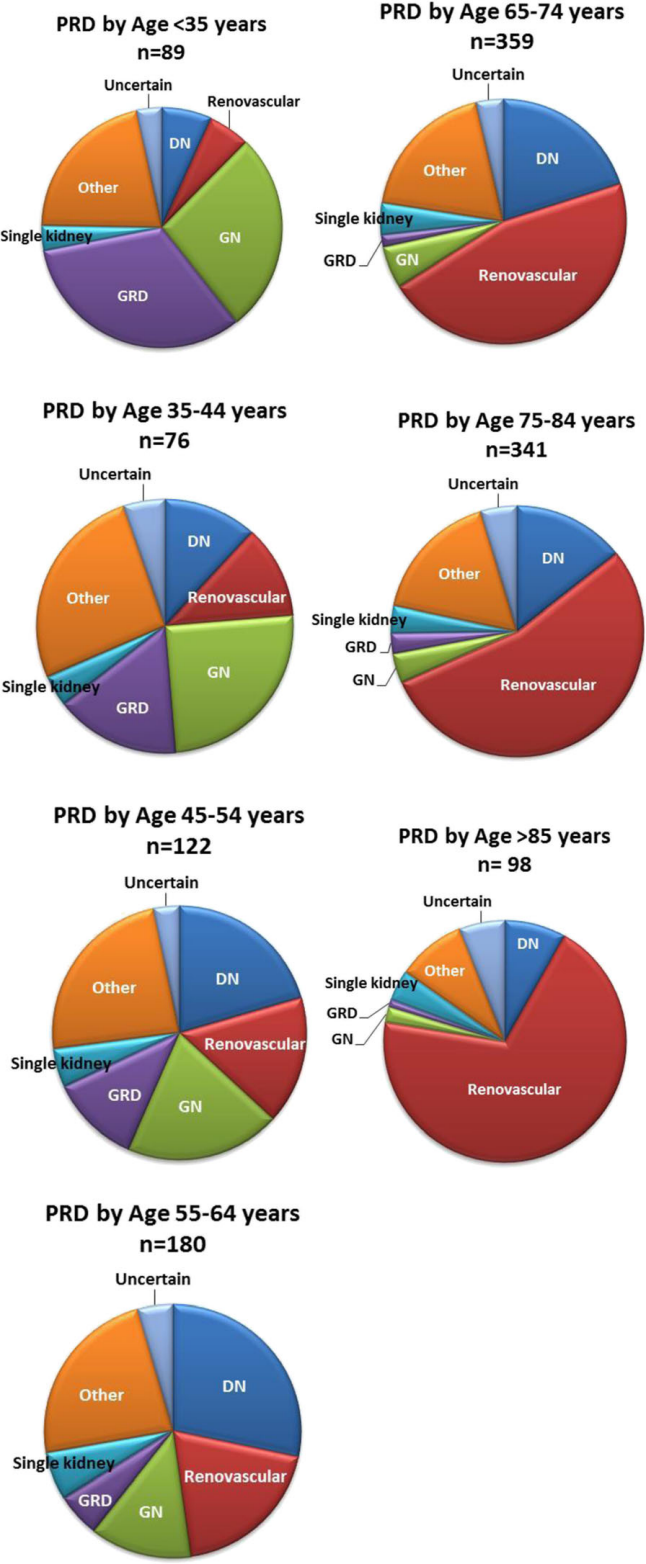
Primary Renal Diagnosis (PRD) in different age groups

Overall, renovascular disease (including hypertension) was the most common primary renal diagnosis (38.4%), reflecting the preponderance (63% >65 years) of older participants. This contrasts with the ESKD population on RRT in Australia according to ANZDATA, where diabetes continues to be the leading cause [18]. The second leading cause was ‘Others’ category (19.3%) which mostly included analgesic nephropathy, nephrolithiasis, reflux nephropathy and lithium related CKD and number of other rare causes. Diabetic nephropathy was the 3rd most common cause of CKD in our study population, with a prevalence of 17.4%. The proportions by primary renal diagnosis differed markedly by age. The main primary renal diagnoses in different age groups are shown in Fig. 1. In the younger age groups (<65 years) glomerulonephritis (GN) and genetic renal disease (GRD) were the most common primary renal diagnoses/etiologies of chronic kidney disease. Diabetic nephropathy had a modal distribution, and progressively increased in prevalence from 6.7% in the <35 to 28.3% in the 55–64 year age group before declining to 8.1% in the 85+ age group. Overall, renovascular disease (including hypertension) progressively increased in prevalence with participant age, reaching a peak of 69.3% in oldest age group (85+).

**Fig. 2:**
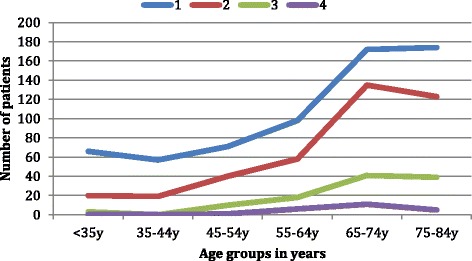
Number of renal disease diagnoses in different age groups

The number of renal disease diagnoses per patient ranged from 1 to 4. In total, 55% of participants had a single renal diagnosis and 33.7%, 9.3% and 2% respectively had 2, 3 and 4 primary renal diagnoses. In <35 year age group, 74.1% of patients had a single renal diagnosis (mean number of diagnoses 1.3, SD 0.5) compared to 58.1% in 85+ age group (mean number of diagnoses 1.5, SD 0.7). Elderly patients (85+ age group) tended to have more than one contributing renal diagnoses (41.8%) attributed to their CKD compared to 25.8% in younger CKD patients (<35 year age group). In the intermediate age group (55-64y) 82 patients (45.5%) had one primary renal diagnosis whilst 98 patients (54.4%) had more than one primary renal diagnosis. Fig. 2 shows the number of renal disease diagnoses [1–4] in different age groups.

### CKD stage at consent to CKD.QLD registry

**Fig. 3:**
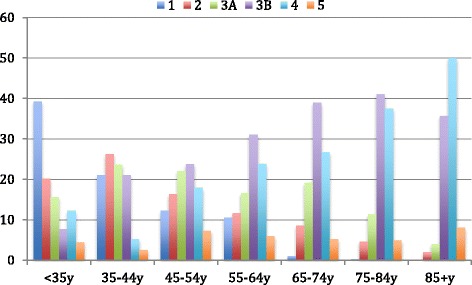
CKD stage at consent in different age groups (y-axis represents percentage of patients)

The distribution of CKD stage differed by age. 74.7% of elderly patients (over 65 years of age) were in later stages of CKD i.e. stage 3B to 5, at the time of enrolment to the CKD.QLD registry. In contrast, 60.3% of younger patients (under 65 years) had CKD stage 1,2 or 3A at the time of consent (Fig. 3).

### Comorbidities

Of leading comorbidities, excluding anaemia and dyslipidaemia, 9 were reviewed, with a mean of 0.7 (SD 0.8) in those <35 years and mean of 2.9 (SD 1.2) in 85+ age group. All comorbidities were more common in males than females; mean 2.8 (SD 1.7) in males as compared to 2.2 (SD 1.5) in females (*p* < 0.001). Comorbidities documented include coronary artery disease (CAD), cerebrovascular disease (CVD), hypertension (HTN), peripheral vascular disease (PVD), diabetes mellitus (DM), gout, sleep apnoea, chronic lung disease and cognitive impairment/dementia.

### Outcomes

#### Start of renal replacement therapy (RRT)

**Fig. 4:**
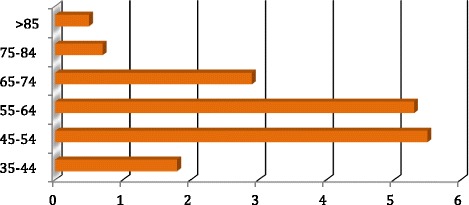
Incidence (rate) of renal replacement therapy per 100 person years

**Fig. 5:**
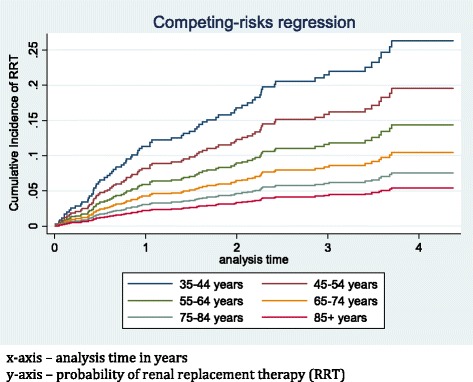
Cumulative incidence of renal replacement therapy considering competing risk

Eighty-six (6.8%) patients started RRT during the follow-up period. Relatively more were male (*n* = 50) than female (*n* = 36). Seventeen patients (3.5%) among the cohort of 486 with reno vascular disease started RRT, whilst 79 (16.3%) patients died in the same group over the follow-up period (p < 0.001). Figure 4 shows rates of RRT by age. Rates of RRT increased from 1.8 per 100 person years (35–44 years) to 5.5 per 100 person years (45–54 years) and subsequently fell to the lowest rate of 0.5 per 100 person years in those over 85 years of age. Cumulative incidence of RRT considering competing risk is shown in Fig. 5.

Similar patterns were observed in both sexes. Patients with diabetic nephropathy had the highest RRT incidence rates (7.8 in males and 5.4 per 100 person years in females) compared to other primary renal diagnoses.

### Deaths

**Fig. 6:**
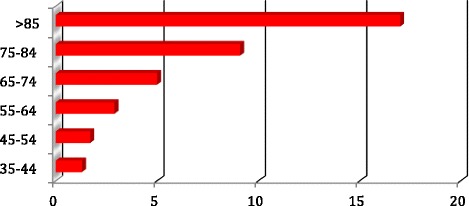
Incidence (rate) of death per 100 person years

**Fig. 7:**
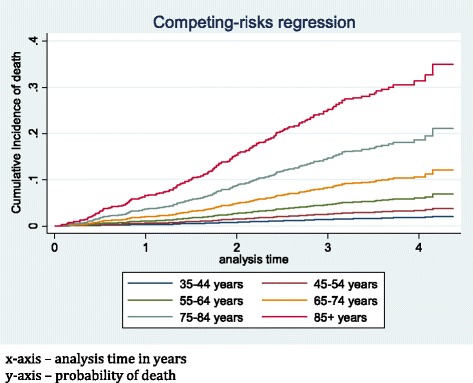
Cumulative incidence of death considering competing risk

One hundred sixty-nine patients (92 males, 77 females) died during the follow-up period in the study. The incidence of death by age group is shown in Fig. 6 below. Death rates progressively increased from 0 per 100 person years in <35 years age group to 17.0 per 100 person years in over 85 s’, with similar trends in males and females. Incidence of death (cumulative) considering competing risk is shown in Fig. 7. Patients with diabetic nephropathy had highest incidence rates of death (7.8 in males and 8.0 in females per 100 person years). Annual progression rate (percentage) from CKD stages 2, 3, 4 and 5 at the time of consent to RRT and death stratified by <75 years and >75 years age groups is shown in Table 2 below.

**Table 2:** Annual progression rates stratified by age

			Progress to RRT		Progress to death	
CKD stage at consent	Number of patients	Follow-up years	Total number of patients progressing to RRT	Annual progression rate (%)^a^	Total number of patients’ death	Annual progression rate (%)^a^
Patients aged <75 years						
CKD 2	110	279.5	1	0.4	2	0.72
CKD 3	406	1087.9	8	0.7	28	2.57
CKD 4	176	396.7	39	9.8	30	7.56
CKD 5	45	47.9	31	64.7	3	6.26
Patients aged >/=75 years						
CKD 2	18	38.4	0	0.0	4	10.4
CKD 3	218	537.3	1	0.2	29	5.4
CKD 4	177	391.5	3	0.8	58	14.8
CKD 5	25	28.3	3	10.6	14	49.5

Notes: Annual rate of progression (per 100 person years) = (total patients (RRT or death) / follow-up years) ^a^ 100

**Table 3:** Hospitalizations in all age groups (poisson regression used to test significance between rates of hospitalizations in different age groups)

Age group	Follow-up years	No. of hospitalizations	Rate of hospitalization per 100 person years	Incidence rate ratio	*p* value
<35y	151	97	64.2	1.0	Reference
35-44y	135.6	79	58.2	0.9	<0.001
45-54y	177.0	171	96.6	1.7	<0.001
55-64y	252.9	262	103.6	1.8	<0.001
65-74y	523.3	394	75.3	1.3	<0.001
75-84y	507.2	632	124.6	2.2	<0.001
85 + y	108.7	97	89.2	1.6	<0.001
Total	1856.1	1732	93.3		

### Progression (change in eGFR over follow-up)

In patients in whom eGFR (calculated by CKD EPI equation) was followed for at least one year (*n* = 905), 19.3% (175/905) had improvement in eGFR (defined by increase of >5 ml/min/year) whilst 24.7% (224/905) experienced significant loss of eGFR (progression) defined by loss of >5 ml/min/year (*p* = 0.006). 66.6% of patients under 35 years and 55.4% of 85+ years had stable renal function (eGFR changes up to +/−5 ml/min/year). Elderly people (85+) were more likely to have progression of CKD (29.2%) as compared to 13.3% of those ages <35 yr. Overall, the majority of the cohort (55.9%) had stable renal function over a 12-month follow-up.

### Hospitalisations

Five hundred fourteen patients (261 males and 253 females) had at least one hospitalisation to Royal Brisbane & Women’s Hospital during the follow-up period, out of total population of 1150. As shown in Table 3, patients over 65 years of age had the highest number of hospitalisation episodes. There were 394 hospitalisation episodes in those aged 65–74 years, 632 episodes in those between 75 and 84 years and 97 episodes in over 85 years. The rate of hospitalisation per 100 person years increased from 64.2 in patients less than 35 years of age to a peak of 124.6 in 75–84 year age group.

## Discussion

Australia has excellent available data regarding End Stage Kidney Disease (ESKD) patients on RRT in the form of the ANZDATA registry [19] but there is paucity of data regarding earlier stages of CKD before the need for RRT, or those who do not go on to RRT. Our study is one of the few providing opportunities to look at pre-terminal CKD, through examining patients referred by other health care providers (mostly in primary care) to renal specialty services, across all age groups from young adults through to very elderly. This study population consists of 1265 patients with CKD referred for evaluation and/or management to a single renal specialty service, although with multiple clinics, in a metropolitan setting (Kidney Health Service (KHS), Metro North Hospital and Health Service (MNHHS)), within the publicly funded Queensland Health system.

There were no exclusions in our study based on age or range of eGFR. This contrasts with other published studies of renal specialty-based CKD patients. Other CKD cohort studies like German Chronic Kidney Disease (GCKD) [20], the Chronic Renal Insufficiency Cohort (CRIC) [21] and the Chronic Kidney Disease Japan Cohort (CKD-JAC) [22] excluded patients over 75 years of age or excluded patients with milder forms of CKD, represented by eGFR over 60 ml/min/1.73m^2^ (GCKD, CKD-JAC and CKD-REIN – The French Chronic Kidney Disease Renal Epidemiology and Information Network cohort study [23]) or over 45 ml/min/1.73m^2^ (CanPREDDICT [24] Cohort profile: Canadian study of prediction of death, dialysis and interim cardiovascular events). The International Network of CKD cohort studies (iNET-CKD) has recently been established to promote research and conduct observational studies of appropriate sample size, follow-up and defined objectives including patient level outcomes. This will facilitate comparative research and identify the differences (genetic, behavioural and health services factors) associated with the course of CKD [25].

Additional strengths of this study include the diversity of subjects, representing the very multicultural mix of the Australian population. In contrast, the GCKD [20] cohort had 100% Caucasian patients, CanPREDDICT [24] had a low prevalence of non-Caucasian individuals and the CKD-JAC cohort [22] had only Japanese patients who have lower cardiovascular disease prevalence. Our cohort also had uniform access to health resources and shared practice protocols within the entire Queensland public health service. Moreover, our study links participant demographics, clinical characteristics and laboratory data to clinically significant outcomes, while some studies e.g. Alberta Kidney Disease Network (AKDN) [26] rely on only laboratory data to describe a study population. The comprehensive nature of our data provides a basis for international comparison and cross-validation of findings.

In this study we found significant heterogeneity by age in terms of CKD stage, etiology and number of renal disease diagnosis, comorbidities as well as outcomes of renal replacement therapy, death, rate of progression of CKD and of hospitalizations. Being sourced from specialty nephrology practices, the majority of patients (66.8%) were in more advanced stages of CKD (stage 3B, 4 and 5) at entry into the CKD.QLD cohort. However, the level of kidney dysfunction at the time of entry was dependent on age. Younger patients entered at earlier stages of CKD, with three quarters (75.2%) younger than 65 years in either Stage 1 or 2. This contrasts with only 24.7% of patients over 65 years entering at stage 1 or 2. This could represent variability in referrers practice. 63% of our patients were ≥65 years old, themselves a heterogeneous population, and 34.7% were ≥75 years old at consent. Better understanding of these individuals is needed, so efforts can be made to slow their disease progression and reduce the burden of ESKD and non-renal complications, because specific therapies to target CKD are very limited as of today, and the elderly are the fastest growing population demographic.

Not surprisingly the prevalence of vascular disease, including renovascular disease, increased from younger to elderly age groups. It was the most common attributable diagnosis in the majority of our patients (38.4%). Interestingly, diabetic nephropathy contributed to only 17.4% of CKD in our cohort in comparison to much higher prevalence in other studies [27] and it varied with age. Older participants tended to have more than one primary renal diagnosis but a significant minority of younger participants also had multiple renal disease diagnoses. The elderly cohort also had a greater burden of co-morbid diseases, which they had presumably accumulated over the course of their lives or might have been facilitated or accelerated by CKD.

The renal function of the majority (55.9%) of participants did not deteriorate over the study interval. Whilst this may represent successful models of care, other explanations include a more benign natural history of CKD than expected: it is noteworthy that these data align with the results of CanPREDICCT study [24]. However CKD did progress in a subgroup. This has implications for CKD prognostication to target those who are more likely to progress.

Not unexpectedly, participants in the older age groups have higher incidence of death and lower incidence of RRT, either dialysis or transplantation. The lower rates of RRT may indicate increasing provision of renal supportive/conservative care as an alternative choice to RRT in those elderly frail patients with multiple medical co-morbidities. Older patients also tended to have more hospitalizations with longer length of stay and utilized more health resources as opposed to younger patients. Tonelli et al. [28] recently demonstrated that comorbidity in CKD patients is an important driver of adverse clinical and economic consequences.

Our study has some limitations. It is a single center study although patients are drawn from multiple hospital and community clinics. As only referred and consented patients with CKD are included in the CKD.QLD registry, therefore, we are unable to generalize our findings to the CKD patients being managed by primary care physicians with no specialist nephrology input. There may be a referral bias as elderly patients with renal impairment are less likely to be referred to specialist renal services than their younger counterparts. Furthermore there are some uncertainties in the etiology of CKD as many physicians made the diagnoses without biopsy confirmation.

## Conclusion

Renovascular disease is the most prevalent attributable cause of CKD overall, reflecting its dominance in the fastest growing elderly (over mean age of 65 years) demographic. Within this population, there is heterogeneity by age in the characteristics and outcomes of CKD. These results support a health provider response that is personalised to the patient and the patient’s health profile. Clearly clinical care needs to be tailored to these facts. Our study contributes to the evidence base for research purposes and also to guide policy development and delivery of health care. It forms a basis for further research that personalized care delivers better health outcomes to people with CKD.
